# Vaccine Efficacy against Malaria by the Combination of Porcine Parvovirus-Like Particles and Vaccinia Virus Vectors Expressing CS of Plasmodium

**DOI:** 10.1371/journal.pone.0034445

**Published:** 2012-04-17

**Authors:** Dolores Rodríguez, Gloria González-Aseguinolaza, Juan R. Rodríguez, Aneesh Vijayan, Magdalena Gherardi, Paloma Rueda, J. Ignacio Casal, Mariano Esteban

**Affiliations:** 1 Department of Molecular and Cellular Biology, Centro Nacional de Biotecnología (CNB), Consejo Superior de Investigaciones Científicas (CSIC), Madrid, Spain; 2 CIMA, Pamplona, Spain; 3 INGENASA, Madrid, Spain; The University of Chicago, United States of America

## Abstract

With the aim to develop an efficient and cost-effective approach to control malaria, we have generated porcine parvovirus-like particles (PPV-VLPs) carrying the CD8^+^ T cell epitope (SYVPSAEQI) of the circumsporozoite (CS) protein from *Plasmodium yoelii* fused to the PPV VP2 capsid protein (PPV-PYCS), and tested in prime/boost protocols with poxvirus vectors for efficacy in a rodent malaria model. As a proof-of concept, we have characterized the anti-CS CD8^+^ T cell response elicited by these hybrid PPV-VLPs in BALB/c mice after immunizations with the protein PPV-PYCS administered alone or in combination with recombinant vaccinia virus (VACV) vectors from the Western Reserve (WR) and modified virus Ankara (MVA) strains expressing the entire *P. yoelii* CS protein. The results of different immunization protocols showed that the combination of PPV-PYCS prime/poxvirus boost was highly immunogenic, inducing specific CD8+ T cell responses to CS resulting in 95% reduction in liver stage parasites two days following sporozoite challenge. In contrast, neither the administration of PPV-PYCS alone nor the immunization with the vectors given in the order poxvirus/VLPs was as effective. The immune profile induced by VLPs/MVA boost was associated with polyfunctional and effector memory CD8+ T cell responses. These findings highlight the use of recombinant parvovirus PPV-PYCS particles as priming agents and poxvirus vectors, like MVA, as booster to enhance specific CD8+ T cell responses to Plasmodium antigens and to control infection. These observations are relevant in the design of T cell-inducing vaccines against malaria.

## Introduction

The development of a malaria vaccine is a major goal in the fight against infectious diseases, as infection with *Plasmodium falciparum* causes an estimated 225 million cases of malaria and 781,000 deaths from the disease worldwide, mostly among children in Africa [1]. Progress in this direction has been obtained with the demonstration in phase 2 clinical trials that the administration of an anti-sporozoite vaccine based on the parasite CS protein fused with the hepatitis B antigen (referred as RTS,S) in combination with the adjuvants AS01 and AS02 generates in children and infants around 50% clinical efficacy against malaria [2], [3], [4] [5]. Immunological analysis indicates that the likely main protective mechanism is the induction of antibodies against the CS protein, while CD8+ T cell responses might play a minor role as they are very modest [4], [6]. The combination of anti-CS antibody concentrations titers and CS-specific TNFα(+) CD4(+) T cells could account for the about 50% level of protection against clinical malaria conferred by RTS,S/AS01(E) [4], [7]. Based on the CS-protective efficacy, a phase III clinical trial is ongoing in 11 centers in Africa with RTS,S (NCT00866619). Thus far, initial results of this trial at 12 months provided about 50% reduction of clinical episodes of malaria and severe malaria in vaccinated children 5 to 17 months of age [8]. The limited protection thus far obtained with the RTS,S vaccine, suggest the need to improve the efficacy of this malaria vaccine.

Because both B and T cell responses might be required for the control of Plasmodium infection, it could be beneficial for the current RTS,S vaccine to also enhance CD8+ T cell responses. Numerous protocols have been developed that trigger specific B and T cell responses to Plasmodium antigens with different degrees of protection in animal models, using a combination of viral and non viral vectors [9]. Among vaccine protocols, the heterologous prime/boost immunization using poxvirus vectors as booster has been shown to be quite effective at inducing T cell responses with protection in animal model systems. In fact, influenza and VACV vectors both expressing the CS antigen of *Plasmodium yoelii* (*P. yoelii*), provided evidence in mice that the combination of influenza/poxvirus vectors was highly effective at inducing specific CD8+ T cell responses and high levels of protection against rodent malaria, and that the order of vector immunization was critical [10]. The question of which are the most appropriate vectors for use in human trials is still unclear and is based on several assessments in which nature of antigens, immune potency, durability, efficacy, safety and cost play important roles. Recently, a combination of poxvirus vectors (fowlpox and MVA) expressing different Plasmodium antigens has shown lack of protection in phase I/IIa clinical trials [11], and this could be related to the complex nature of the six pre-erythrocytic malaria antigens linked together in a single protein. The use of single protein components as immunizing agents that can prime an anti-malaria immune response which is expanded after a boost with a poxvirus vector could be a favorable option to avoid antigen competition. Proteins can be delivered either alone, conjugated with adjuvants, in the form of fusion polypeptides or as part of virus-like particles (VLPs).

The use of VLPs might be preferable due to the ease of production and of presentation of the malaria antigen, as the VLPs behave as native virus particles during entry and processing into antigen-presenting cells. It has been previously demonstrated that chimeric PPV-VLPs carrying heterologous epitopes, when injected intraperitoneally (i.p) into mice, activate strong CD4+ and CD8+ T-cell responses specific for the foreign epitopes, and these responses are mediated by dendritic (DC) cells and influenced by the flanking sequences [12], [13], [14]. PPV-VLPs are formed by the assembly of 60 copies of the major virus capsid protein (VP2) of PPV [15]. PPV-VLPs have been engineered to deliver CD8+ T-cell epitopes [12] or CD4+ T cell helper epitopes [16] inserted into the N terminus of the PPV VP2. Cytotoxic T lymphocyte (CTL) response was characterized by a high frequency of specific T cells of high avidity [17]. Moreover, the CTL activation does not require CD4+ T cell help [12]. PPV-VLPs are captured by DC through macropinocytosis and these cells are the only antigen presenting cells (APCs) for PPV-VLPs [13]. VLPs can be found in the endosome of DC, where processing of the epitopes inserted in the VLPs takes place [13]. In addition, B cell epitopes can be engineered on the surface of the parvovirus VLPs by manipulating the VP2 loops to elicit a potent antibody response [18].

Thus, to enhance the CD8+ T cell response of a CS-based immunization procedure, in this investigation we compared in mice the immunogenicity and anti-malaria efficacy of protocols based on priming with parvovirus PPV-VLPs carrying a CD8+ T-cell epitope from the *P. yoelii* CS protein, restricted by the H-2K^d^ molecule (SYVPSAEQI) and boosted with either a recombinant replication competent (WR strain) or replication restricted (MVA strain) of VACV, both vectors expressing the entire *P. yoelii* CS protein.

## Results

### The CS-T cell priming effect induced by PPV-PYCS VLPs is markedly enhanced by boosting with a replication competent VACV vector

**Figure 1:**
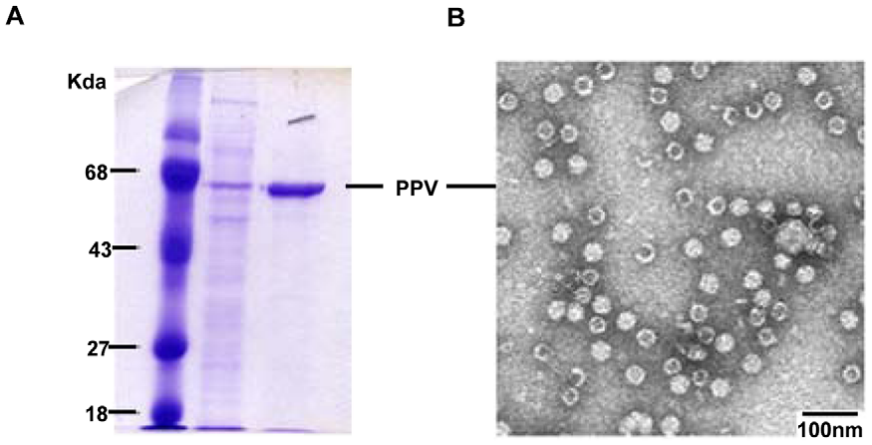
Characterization of PPV-PYCS VLPs. Conditions for generation of the PPV vector expressing the specific CD8+ T cell epitope of *P. yoelii* CS, growth in baculovirus-infected insect cells and purification of the PPV-VLPs is described under Materials and Methods. (**A**). Proteins were resolved by 9% SDS-PAGE and visualized after coomassie-blue staining. Molecular masses of standard proteins (lane 1) are indicated at the left. Lane 2, shows partial purification and lane 3, purified protein with the size corresponding to the PPV VP2. (**B**). Electron microscope image of negatively stained PPV-PYCS VLPs.

**Figure 2:**
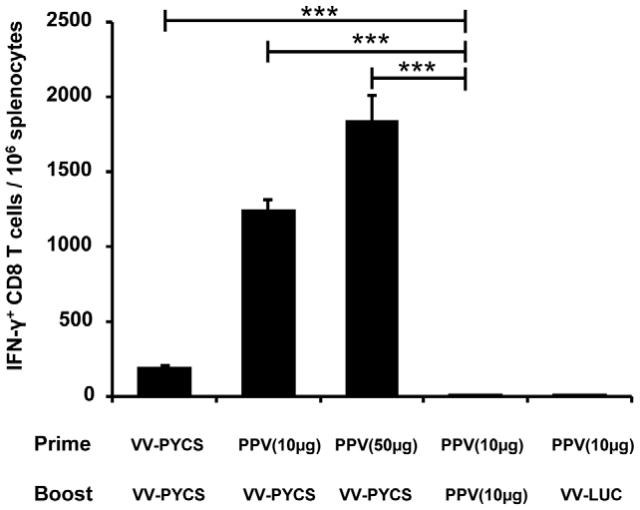
The CS-specific CD8+ T-cell priming effect of PPV-PYCS is markedly enhanced by boosting with replication competent VACV vector expressing CS. Different groups of BALB/c mice (5 per group) were immunized s.c with 10 or 50 µg of PPV-PYCS [PPV(10 µg) and PPV(50 µg) respectively] or with VACV vectors in prime/boost protocols. Fourteen days after the boost CS-specific IFN-γ secreting cells for the plasmodial epitope SYVPSAEQI in splenocytes were measured by ELISPOT as described under Materials and Methods. The results are expressed as the mean of triplicate assays using cultured pooled splenocytes. Statistical values were determined by one-way ANOVA; P values, *P<0.05, **P<0.01, ***P<0.001.

Recombinant PPV-VLPs have been shown to be effective delivery systems of heterologous sequences that trigger specific Th1 type of immune responses to the foreign epitopes when inoculated in animal models [12], [16]. With the aim to define the priming capacity of PPV-VLPs in prime/boost combination of immunogens, we generated recombinant PPV-VLPs by the insertion into PPV VP2 of the specific CD8+ T cell epitope (SYVPSAEQI) from *P. yoelii* CS protein, restricted by the H-2K^d^ molecule [10]. A DNA fragment coding for the CS epitope was fused to the 5′-end of the gene coding for PPV VP2 capsid protein. This chimeric gene, when expressed by the baculovirus system, produces a CS epitope-VP2 fusion protein that self assembles generating PPV-PYCS VLPs that can be easily purified (Figure 1). Production and purification of PPV-PYCS from baculovirus infected insect cells was performed as described under Materials and Methods. Figure 1A shows a coomassie blue stained SDS-PAGE of the purified PPV-PYCS. A main single band of about 67 kDa was observed on the gel, indicating high purity of the recombinant protein. This was also confirmed by electron microscopy as shown by a photomicrograph of the purified VLPs in Figure 1B. To study the ability of PPV-PYCS to induce a specific anti-CS T cell immune response, and to test whether the achieved immune response could be enhanced after booster with a replication competent VACV recombinant from WR strain expressing the complete CS protein (VV-PYCS), we immunized groups of BALB/c mice (5 per group) with PPV-PYCS given subcutaneously (s.c) (10 or 50 µg/ per mouse), and boosted 14 days later with 10^7^ pfu/mouse of VV-PYCS by the same route. At 14 days after the booster, splenocytes from immunized animals were subjected to a standardized and quantitative ELISPOT assay, measuring IFN-γ secreting cells [19]. We used MHC-class I P815 cells (H-2^d^) as APC, pulsed with 10^−6^ M of the synthetic peptide SYVPSAEQI, corresponding to the *P. yoelii* CS protein. Previous studies have shown the CD8+ T cell responses of the *P. yoelii* CS-peptide (SYVPSAEQI), which is specific for IFN-γ secreting cells [10] [20]. As shown in Figure 2, animals immunized with two doses of 10 μg of PPV-PYCS did not develop a significant CS-specific CD8 T cell response. However, when animals were boosted with a VV-PYCS a significant (*p*<0.001; one-way ANOVA) increase in the response was observed. This increment was dose-dependent, since animals primed with the highest dose of PPV-PYCS (50 µg) developed the strongest response. In addition, this response was specific, as a non-related recombinant VACV expressing luciferase when used for booster (VV-LUC) had no effect on T cell responses. These findings revealed that while PPV-PYCS *per se* is low inducer of IFN-γ secreting cells, however, is an effective priming component when booster is done with the replication competent VV-PYCS recombinant vector.

### Priming with PPV-PYCS followed by booster with replication competent VV-PYCS protects mice against liver stage parasites after challenge with *P. yoelii*

**Figure 3:**
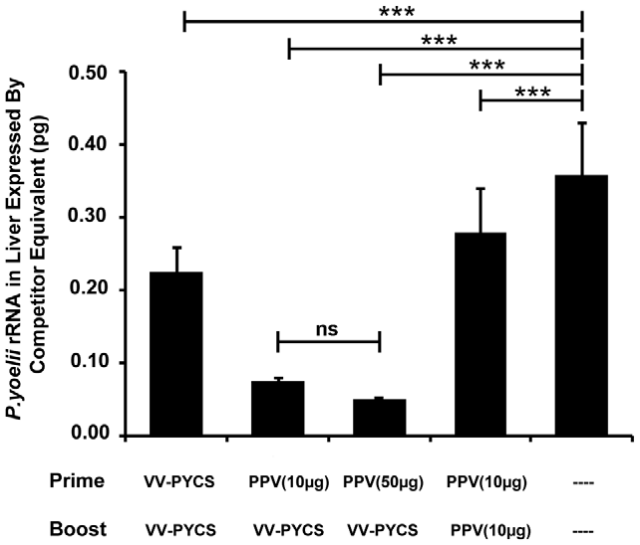
Protective capacity of PPV-PYCS prime followed by booster with replication competent VACV vector expressing CS. Groups of BALB/c mice (5 per group) were immunized with different amounts of PPV-PYCS [PPV(10 µg) and PPV(50 µg)] or non-vaccinated and two weeks later animals were boosted with the replication competent VACV vector from the WR strain (referred as VV-PYCS) or with PPV-PYCS. Thirteen days later, animals were challenged with sporozoites and the amount of parasite rRNA in the liver of each animal was estimated 42 h after the challenge, as described under Materials and Methods. Results are expressed as the mean with standard deviation. Statistical values were determined by one-way ANOVA; *P* values, *P<0.05, **P<0.01, ***P<0.001. Lane (–), non-vaccinated.

To determine whether PPV-PYCS prime/VV-PYCS boost immunization could induce protective immunity against pre-erythrocytic stages of the parasite, BALB/c mice (5 per group) were immunized with 10 or 50 μg of PPV-PYCS administered s.c and 2 weeks later were boosted with one dose of 10^7^ pfu of VV-PYCS administered by the s.c route. Thereafter, immunized mice were challenged i.v with 5x10^5^ *P. yoelii* highly infective sporozoites. Parasite development was monitored at 42 h after challenge by measuring plasmodial rRNA in the liver of the challenged mice by semi-quantitative RT-PCR assay, a well defined and standardized protocol to evaluate protection [10], [21]. We found that mice immunized following the prime-boost immunization scheme had very low levels of the parasite load in the liver, with an 86% percent reduction when the dose of PPV-PYCS was 50 μg and 81% reduction in parasitemia with a dose of 10 μg (Figure 3; *p*<0.001 by one-way ANOVA). Mice immunized with either two doses of PPV-PYCS or VV-PYCS showed a partial decrease of the parasite load in the liver in comparison with non-vaccinated control mice. These findings showed that administration of PPV-VLPs alone does not protect against sporozoite challenge while the combination of PPV-VLPs/VACV is an effective protocol to control *P. yoelii* infection.

### Booster with a replication-restricted MVA-PYCS vector triggers strong CD8+ T cell responses to CS after priming with PPV-PYCS

**Figure 4:**
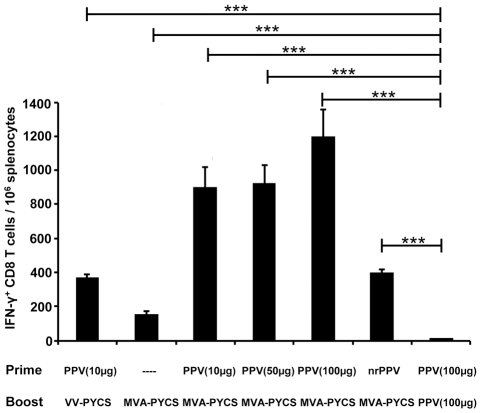
Booster with replication restricted MVA-PYCS vector triggers strong CS CD8+ T-cell response after priming with PPV-PYCS. Groups of BALB/c mice (5 per group) were immunized s.c with different amounts of PPV-PYCS [PPV(10 µg), PPV(50 µg) PPV(100 µg)], with non-recombinant PPV-VLPs (nrPPV) or with VACV vectors (VV-PYCS or MVA-PYCS) in prime/boost protocols and 14 days after the boost CS-specifc IFN-γ secreting cells for the plasmodial epitope SYVPSAEQI in splenocytes were measured by ELISPOT as described under Materials and Methods. Purified PPV-VLPs without the peptide insert were used as control. The results are expressed as the means of assay triplicates of cultured pooled mouse splenocytes. Statistical values were determined by one-way ANOVA; P values, *P<0.05, **P<0.01, ***P<0.001.

While studies shown in Figures 2 and 3 were performed with replication competent VACV, it was of interest to know to what extent the use of a replication restricted and highly attenuated vector like MVA [22] could boost PPV based anti-malaria immune response. Thus, groups of BALB/c mice (5 per group) were primed s.c with different doses of PPV-PYCS (10, 50 and 100 μg) and two weeks later those mice were boosted s.c with 10^7^ pfu of the attenuated MVA-PYCS vector. A group of mice immunized with 50 µg of PPV-PYCS and boosted with 10^7^ pfu of VV-PYCS was included as comparison for replication competent vector. Also groups of mice primed/boosted with 100 µg of PPV-PYCS, or primed with PPV-VLPs lacking the CS-specific CD8+ T cell epitope and boosted with MVA-PYCS were included for reference. The frequencies of *ex vivo* splenocytes producing IFN-γ upon MHC class-I restricted peptide stimulation was determined by ELISPOT assay two weeks later. As shown in Figure 4, the groups of animals receiving PPV-PYCS/MVA-PYCS had the highest values of IFN-γ secreting cells (*p*<0.001 by one-way ANOVA). These values were even higher than those observed in the group PPV-PYCS/VV-PYCS. Significantly, a low immune response was obtained when single dose of MVA-PYCS was used, or when it was given after priming with non-recombinant VLPs. In addition, a minor immune response was obtained when PPV-PYCS was used at 100 µg/per dose (Figure 4).

These findings clearly demonstrate that priming with PPV-PYCS is necessary to activate specific T cells, but to obtain large expansion of these T cells it required MVA-PYCS boost.

### Protection against parasites after prime/boost with PPV-PYCS/MVA-PYCS protocol

**Figure 5:**
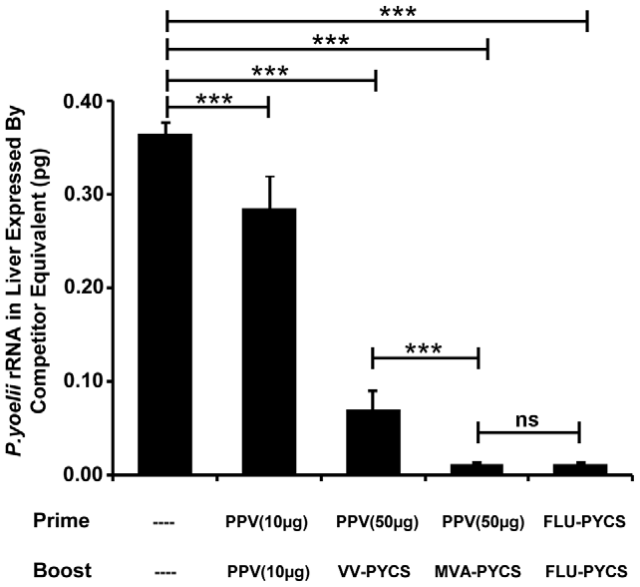
Protection against malaria after prime/boost with PPV-PYCS/MVA-PYCS protocol. Groups of BALB/c mice (5 per group) were immunized with different amounts of PPV-VLPs [PPV(10 µg) and PPV(50 µg)] and two weeks later animals were boosted with the replication restricted MVA-PYCS or the replication competent VV-PYCS. A positive control group primed with an influenza virus recombinant expressing the CD8+ T cell epitope and booster with VV-PYCS, a protocol previously shown to induce high protection against the parasite, was included [10]. Thirteen days later, animals were challenged with sporozoites and the amount of parasite rRNA in the liver of each animal was estimated 42 h after the challenge, as described under Materials and Methods. Results are expressed as the mean with standard deviation. Statistical values were determined by one-way ANOVA; *P* values, *P<0.05, **P<0.01, ***P<0.001.

Next we examined if the increase in immune response observed after MVA-PYCS boost is translated into a high degree of protection. In this experiment we compared the protective immunity elicited against parasite challenge after prime-boost immunization with PPV-PYCS followed by VV-PYCS or MVA-PYCS, with that obtained in a group of mice immunized with a recombinant influenza virus expressing *P. yoelii* CS-CD8+ T cell epitope (Flu-PYCS) and boosted with VV-PYCS. As shown in Figure 5 the degree of protection, based on reduced parasitic burden in the liver, was higher in the group of mice primed with PPV-PYCS and boosted with MVA-PYCS than in the group of mice boosted with VV-PYCS (*p*<0.001 by one-way ANOVA. On the other hand the 97% inhibition of parasite development in the group of mice boosted with MVA was similar to that obtained in the group of mice immunized with Flu-PYCS/VV-PYCS which serves as a reference, as it is a well established and standardized protocol previously described to induce high level protection after parasite challenge [10]. These findings demonstrate the high degree of protection against liver stage parasites obtained by a PPV/MVA regime.

### PPV-PYCS priming followed by MVA-PYCS boost improved the polyfunctionality and magnitude of memory CD8 T-cell responses

**Figure 6:**
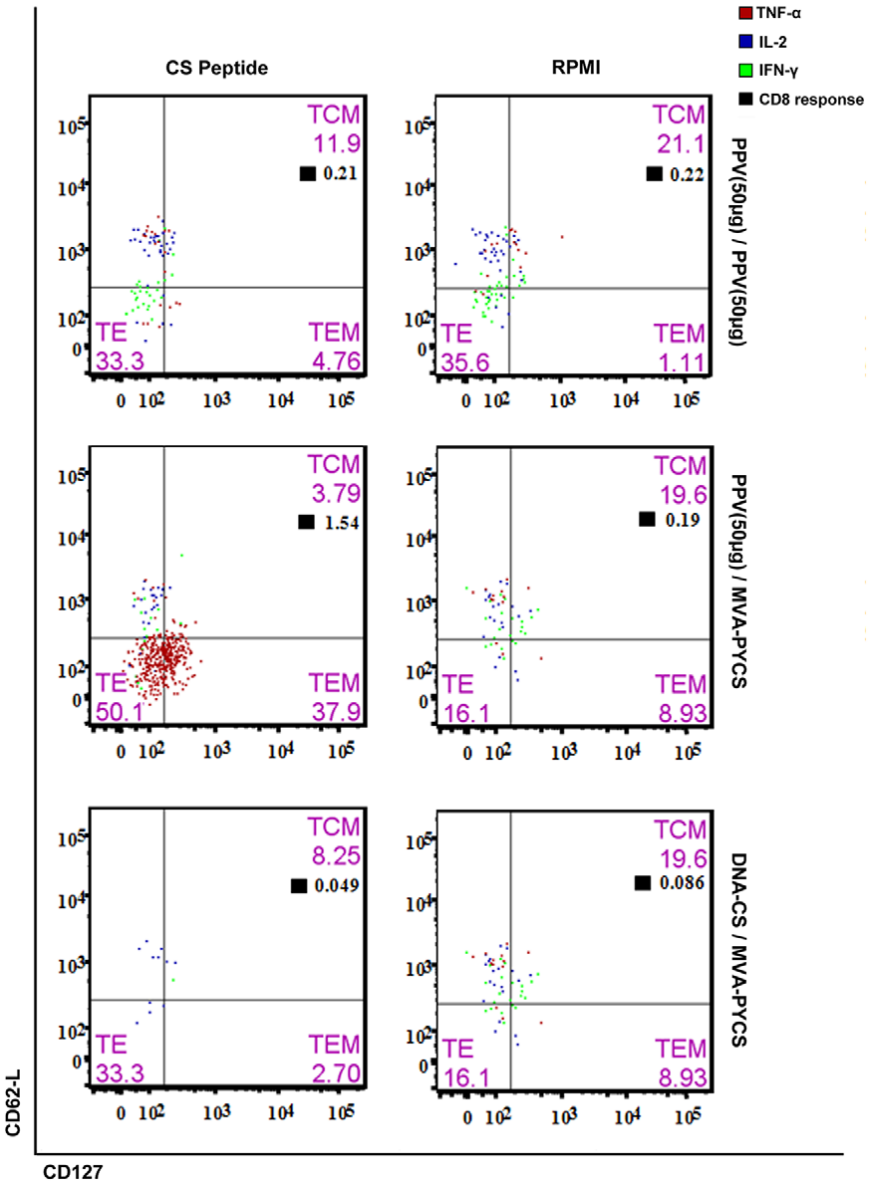
PPV-PYCS/MVA-PYCS prime/boost improves cytokine secretion by memory CD8^+^ T-cells. Groups of BALB/c mice (4 per group) were immunized in prime/boost as indicated in Table 1 and 53 days post boost splenocytes were processed for ICS as described under Materials and Methods. Phenotypic differentiation of CD8^+^ T-cells based on memory markers CD127 (V450) and CD62L (FITC). Each quadrant represents different memory population of CD8 cells with its respective percentages. The distribution of antigen specific CD8+ T-cells secreting cytokines in response to CS peptide stimulation, within the different memory population is also shown. The total CD8+ T-cell response is indicated by the black boxes. The boxes on left indicate the responses towards CS peptide stimulation while the ones on right represent its respective RPMI controls.

**Figure 7:**
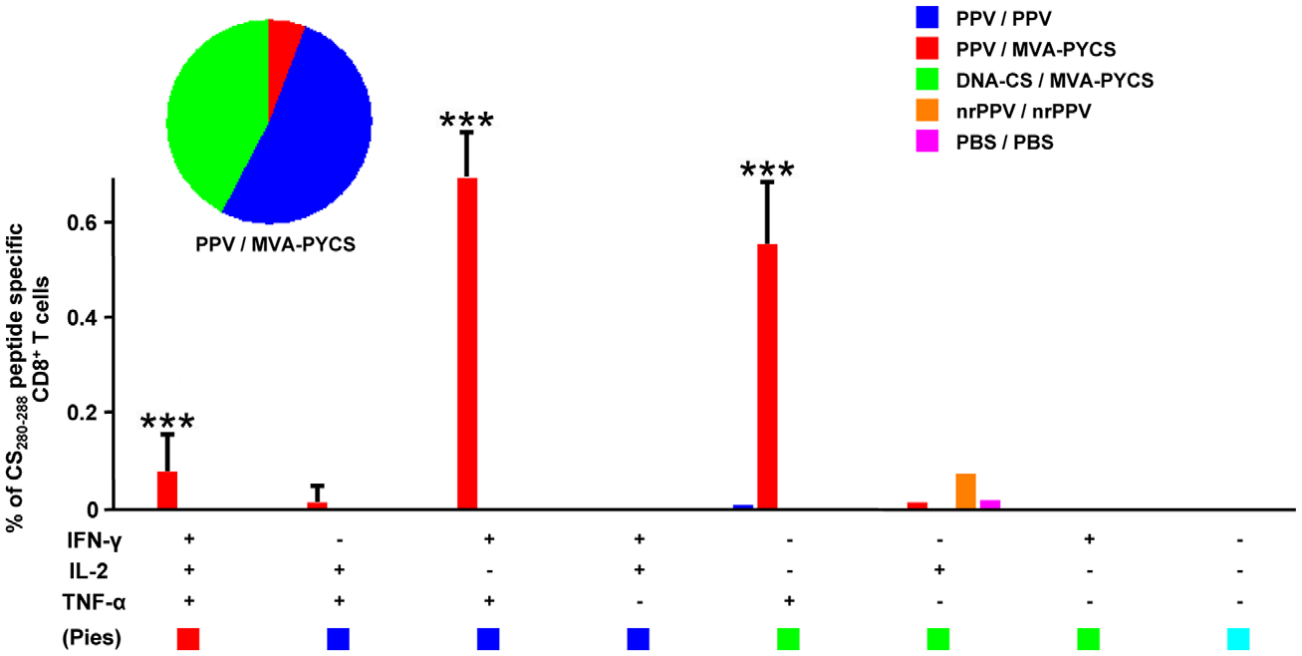
Polyfunctionality of CD8+ T-cells is improved by heterologous prime/boost. Groups of BALB/c mice (4 per group) were immunized in prime/boost as indicated in Table 1 and 53 days post boost splenocytes were processed for ICS as described under Materials and Methods. Memory CD8 T-cells were differentiated into single, double or triple positive cells secreting IFN-γ (PeCy7), TNF-α (PE) and IL-2 (APC). Polyfunctionality is indicated by the pie chart.The data was then analyzed using SPICE software. Data are expressed as mean ± s.e. (n = 4 mice per group). Statistical values were determined by a novel approach previously described (33). P values were calculated between PPV/MVA-PYCS and PPV/PPV groups. **p<0.01; ***p<0.001.

Enhancement in the quality and magnitude of memory CD8+ T-cell response is known to correlate with better protection in malaria [23], [24]. Therefore, to define the CD8+ T cell responses induced by VLP-PYCS/MVA-PYCS prime-boost, we performed intracellular cytokine staining (ICS) study with splenocytes obtained from mice immunized by various protocols and collected 53 days post-boost to evaluate memory T cells. The vaccination regime based on VLP-PYCS prime/MVA-PYCS boost generated a robust response in mice when compared to VLP-PYCS prime/boost or DNA-PYCS/MVA-PYCS prime/boost, as revealed after separation of CD8 T-cells based on memory markers CD127 and CD62L (Figure 6). This approach helped to differentiate T_Effector_; T_E_ (CD127^Lo^CD62L^Lo^), T_Effector Memory_; T_EM_ (CD127^Hi^CD62L^Lo^) and T_Central Memory_; T_CM_ (CD127^Hi^CD62L^Hi^). Considering the negligible CD8+ T-cell responses generated when using DNA-PYCS as priming agent, a VLP-PYCS/MVA-PYCS vaccine regime elevated the CS specific CD8+ T cell response by 1.5 fold (*p*<0.001 determined as in ref 33) (Fig 6). Interestingly, the majority of the CD8+ T cells, following stimulation, rapidly acquired T_E_ phenotype. However a significant increase in the T_EM_ population was also observed (Fig 6). Furthermore, the polyfunctional nature of the CD8 T-cells generated, suggested the enhanced capability of VLP priming followed by VACV boost. The majority of the CD8 cells produced were double positive for IFN-γ and TNF-α (IFN-γ^+^TNF-α^+^) (*p*<0.001) (Figure 7). In addition, there was high surge in the TNF-α secreting cells (*p*<0.005). Although, at lower levels, there was a significant induction of triple positive cytokine secreting cells (IFN-γ^+^TNF-α^+^IL-2^+^) (*p*<0.001). Thus, the data revealed how VLP-PYCS prime/MVA-PYCS boost improves the polyfunctionality and the population of memory CD8+ T cells in immunized mice, immune parameters which might be relevant in protection against malaria.

## Discussion

While one of the main interests in the malaria vaccine field is to develop immunogens based on proteins for safety considerations, however, the immune response triggered by proteins is generally weak with a bias for Th2 type. Thus, efforts have been directed to combine proteins with adjuvants and immunostimulatory molecules, to enhance specific innate immune responses and to drive the response to a Th1 type. In fact, the current phase III clinical trial with malaria vaccine RTS,S is based on CS protein fused to hepatitis B antigen and combined with a potent adjuvant AS01. Because previous studies with the RTS,S vaccine have shown weak CD8+ T cell responses in vaccinees, in this investigation we have asked the question as to what extent CS antigen presentation by chimeric VLPs obtained from PPV is able to prime CD8+ T cell responses, and how these responses can be enhanced when combined with poxvirus vectors expressing CS. In addition, we have asked in which grade specific CD8+ T cell immune responses are associated with protection against malaria and what are the CD8+ T cell populations induced. This proof-of-concept has been tested in mice. In this work we have generated recombinant parvovirus-derived VLPs expressing the CTL epitope from the *P. yoelii* CS protein.

The PPV-VLP system presents several advantages such as the long term stability of VLPs in addition to high levels of protein expression. Although the system has not been rigorously tested for the largest insert size that can be accommodated without disrupting the particle, there would be two ways to overcome the limitation of the system to accept large insertions. First, it is relatively simple to build different PPV-VLPs containing different CD8+ epitopes that can be mixed during administration. Second, PPV VP2 containing different epitopes can be co-expressed in the baculovirus system to prepare VLPs with mixtures of different epitopes. These possibilities would facilitate the use of multiple CD8+ epitopes, as required for human vaccines, or their combination with CD4+ or B cell epitopes. These combinations should result in a more efficient vaccine design.

Groups of mice immunized in a prime/boost regimen with two doses of PPV-PYCS did not develop significant anti-CS cellular immune response, determined by the ELISPOT assay. However, when animals were immunized with different doses of PPV-PYCS, followed by the recombinant VV-PYCS, expressing the full CS protein from *P. yoelii*, a strong CD8^+^ T cell response was generated against this antigen. This response was about 10 times higher that the one obtained with two doses of VV-PYCS. When we used for priming two different doses (10 and 50 µg) of PPV-PYCS followed by a booster with 10^7^ PFU of VV-PYCS there was a dose response effect, obtaining the highest CD8+ T cell response with the highest dose of PPV-PYCS. In this experiment we observed again that this response was much higher than that obtained with two doses of VV-PYCS. We have also included as control a non-related recombinant VACV for booster, which showed that the CD8+ T cell response primed by the recombinant VLPs is boosted in an antigen dependent manner, and cannot be boosted by a non-related VACV recombinant.

Importantly, when we analyzed protection after challenge with the parasite in animals immunized as described above, quantified as reduction in the amount of Plasmodium rRNA in the liver with respect to non-immunized animals, we observed maximal protection in mice primed with PPV-PYCS followed by a booster with VV-PYCS or MVA-PYCS. Because safety is a major concern for developing poxvirus based vaccines, we included for boosting a recombinant vector expressing the full-length CS protein based on the highly attenuated MVA strain instead of the recombinant based on replicating WR strain. When mice were immunized with 10, 50 or 100 µg of PPV-PYCS followed by the MVA recombinant (10^7^ pfu), we obtained a stronger cellular immune response that was about 2 to 3 times higher than that obtained when we used for boosting a recombinant based on the WR strain. While two doses of PPV-PYCS did not elicit significant cellular immune responses, these PPV-VLPs were able to prime a specific anti-CS CD8+ T cell response that could be subsequently be boosted by MVA-PYCS. When PPV-PYCS was substituted by PPV-VLPs without the CS epitope during priming, the magnitude of the response after booster with the virus was similar to that obtained with only one dose of MVA-PYCS.

The magnitude of the specific cellular immune response was associated with protection, defined by markedly reduced parasites in the liver two days after challenge. Thus, when groups of mice immunized as before were challenged with the Plasmodium parasite, maximal protection was observed in animals boosted with the MVA-PYCS recombinant. The high protection (about 95%) obtained after priming with PPV-PYCS and boosting with MVA-PYCS was comparable to that obtained after immunization in similar prime/boost approach but using instead two live recombinant viral vectors, i.e influenza expressing the CD8^+^ T cell epitope followed by VV-PYCS, that we have previously reported to be protective [10]. This is an important consideration, since by using PPV-VLPs and MVA immunogens we are able to achieve similar protective immune response than previously attained with two replicating viral vectors.

How can we explain the enhanced response obtained by PPV-VLPs/poxvirus prime/boost approach and its association with protection against the malaria parasite? Based on current understanding of prime/boost protocols, and due to the induction of anti-vector immunity by the VACV prime, we suggest that during priming with PPV-PYCS the particles which are able to enter the cells, are processed by antigen presenting cells and trigger an innate immune response that activate T cells; after booster the primed T cells are expanded as a result of infection of antigen presenting cells by MVA-PYCS and cross-priming effects by induction of apoptosis [25]. In addition, the innate immune response triggered by MVA [26] can also be responsible for T cell expansion. Significantly, the T cell responses observed in the two protocols PPV/VV and PPV/MVA were associated with high levels of protection against the parasite, while low levels of T cells were ineffective to control the parasite. While it is difficult to compare immune correlates between studies performed with different vectors, the magnitude of CD8+ T cell responses after the PPV-VLPs prime/pox boost and degree of protection against the parasite in this study are remarkable. In fact, analysis of the memory CD8+ T cells after prime/boost revealed that the protocol VLP/MVA induced a polyfunctional response with activation of effector memory CD8+ T cells, both immune parameters that could be relevant in protection against malaria [24].

While a number of different prime/boost combination of vectors expressing malaria antigens have shown activation of CD8+ T cell responses and different degrees of protection against murine malaria [9], [27], [28] there are several advantages in the use of PPV-VLPs and MVA vectors expressing the CS antigen for T cell activation. First, CS has shown good immunogenicity profile with efficacy in preclinical assays whether from DNA, Ty-particles, a fusion protein or delivered by viral vectors, but most importantly when fused with hepatitis B antigen plus an adjuvant it induced significant protection in children exposed to malaria, indicating the CS protective capacity if formulated properly for immune B and T cell activation. Second, expression of CS as part of PPV-VLPs is able to prime the antigen presenting cell in a way that further booster with the poxvirus vector MVA triggered high levels of CD8+ T cells, which are polyfunctional and of effector memory phenotype, that are associated with high degree of protection after challenge when evaluated by liver stage parasite inhibition. Third, both vectors PPV-VLPs [29] and MVA recombinants [22] can be easily grown to large scale for vaccination purposes. Future studies on potential clinical applicability of the the PPV-VLP platform will require to define if these capsids are capable of carrying large regions of antigen or carrying multiple T-cell epitopes which diverse humans will respond.

Overall, the results reported here demonstrate that prime/boost immunization with PPV-VLPs and MVA expressing CS is a logical vaccine approach to optimize cellular immunity and protection against malaria. These findings are relevant in the design of vaccine strategies against Plasmodium.

## Materials and Methods

### Ethics Statement

The animal studies were approved by the Ethical Committee of Animal Experimentation (CEEA-CNB) of Centro Nacional de Biotecnologia (CNB-CSIC, Madrid, Spain) in accordance with national and international guidelines and with the Royal Decree (RD 1201/2005), permit number: 130/07.

### Cells

African green monkey kidney cells (BSC-40; ATCC CRL-2761) were grown in Dulbecco's modified Eagle's medium (DMEM) supplemented with 10% newborn calf serum (NCS). Chick embryo fibroblast (CEF) cells were obtained from sterile pathogen-free eggs provided by INTERVET (Salamanca, Spain), and primary cultures were prepared according to standard procedures and maintained in DMEM supplemented with 10% fetal calf serum (FCS) (Gibco®, Grand Island, NY, USA). P815 (ATCC TIB-64) mastocytoma cells that express MHC class I molecules H-2d were cultured in DMEM containing 10% FCS.

*Spodoptera frugiperda* clone 9 (Sf9, CRL 1711, ATCC) cells were grown and maintained in suspension at 27°C using Grace's insect tissue culture medium (Gibco®, Grand Island, NY, USA) supplemented with 10% FCS, 0.2% Pluronic F-68 (Sigma-Aldrich, Steinheim, Germany) and antibiotics.

### Growth and purification of VACV expressing the CS protein from *P. yoelii*

The recombinant VACV virus VV-PYCS carrying in the TK locus the entire CS gene of *P. yoelii*, has been previously described [10]. To generate the recombinant virus MVA-PYCS, the CS gene was inserted by homologous recombination into the TK region of the genome of the MVA strain, by using the VV transfer plasmid pSC-PYCS containing the CS DNA sequence under the control of the VACV p7.5 promoter. β-galactosidase-producing plaques were picked, cloned three times, and amplified in CEF cells as previously described [21]. VV-PYCS and MVA-PYCS were propagated and titrated in monkey BSC-40 and CEF cells respectively [30]. Both viruses were purified by banding in sucrose gradients, as previously described [31].

### Construction of a recombinant baculovirus expressing PPV-PYCS

Oligonucleotide 5′-TCGAGATGTCATACGTTCCCTCGGCCGAACAAATCC-3′ and its complementary 5′-TCGAGGATTTGTTCGGCCGAGGGAACGTATGACATC-3′ were designed in order to regenerate the PYCS CD8 epitope SYVPSAEQI plus an initiation codon and two cohesive *XhoI* sites. The oligonucleotides were phosphorylated with T4 polynucleotide kinase, annealed at 70°C for 15 min and ligated into *XhoI*-digested pPPV29mod, which contains a unique XhoI restriction site immediately downstream of the initiation codon of the PPV VP2 gene, as previously described [12]. Then, the chimeric VP2 sequence containing the PYCS epitope was isolated by *BamHI* digestion and subcloned into *BamHI*-digested pAcYM1. The recombinant baculovirus transfer vector was called pAcYM1-PPV-PYCS.

The recombinant viruses were obtained by cotransfection of *Spodoptera frugiperda* (Sf9) insect cells with a mixture of 2 µg of purified transfer vector DNA plus 500 ng of parental BacPAK6 baculovirus DNA, previously linearized with *Bsu36I*, in the presence of cationic liposomes [18]. Transfected cultures were collected when the cells started to show signs of infection, usually 5–6 days later. Recombinant baculoviruses were plaque-purified in the presence of X-Gal (5-bromo-4-chloro-3-indolyl-b-D-galactopyranoside) until no more blue plaques (wild-type) were detected. Then, high-titers stocks (>10^8^ pfu/ml) of the recombinant baculovirus AcPPV-PYCS were obtained.

### Characterization and purification of PPV-PYCS VLPs

Sf9 cells were infected with AcPPV-PYCS at a multiplicity of infection of 0.5 plaque-forming units (pfu) per cell. Cells were collected at 72 h post-infection with a clear cytophatic effect. Purification of PPV-VLPs was carried out as previously described [12], [16]. Briefly, infected Sf9 cells were lysed by hypotonic shock with 25 mM bicarbonate solution at 4°C. Cell debris was removed by centrifugation and the PPV-VLPs present in the supernatant were precipitated with 20% ammonium sulfate, resuspended in PBS and dialysed. The purity of the preparation of PPV-PYCS VLPs was confirmed by sodium dodecyl sulfate polyacrylamide gel electrophoresis (SDS-PAGE). Protein concentration was estimated by densitometric assay with the Bio-Rad protein assay reagent, and using BSA as reference.

Recombinant baculovirus, AcPPV-PYCS, which express under the polyhedrin promoter the PPV VP2 capsid protein fused at its amino-terminus with the PYCS CD8+ epitope, was used for antigen preparation.

### Plasmid construction

#### pCI-Neo-CS

The gene coding for *P. yoelii* CS protein was amplified from MVA-PYCS using the primers CS-XhoI-F (5′-ACTTA*CTCGAG*ATGTGTTACAATGAAGAAAATG-3′) and CS-NotI-R (5′-ATT*GCGGCCGC*TTTAAAATATACTTGAAC-3′) to yield a 972 bp fragment lacking the N-terminal signal sequence and C-terminal GPI sequence. The gene was inserted into a mammalian expression vector, pCI-Neo, that had been previously digested with *Xho*I and *Not*I followed by SAP treatment (Shrimp Alkaline Phosphatase). The CS gene in both the virus and plasmid were sequenced (Secugen; Spain). The plasmid was purified using Qiagen Mega Prep Kit according to manufacturer's protocol. Expression of CS from pCI-Neo-CS was confirmed by transfecting DF-1 cells followed by western blot analysis with CS specific antibodies.

### Parasites

*P. yoelii* (17XNL strain) was maintained by alternating passages in *Anopheles stephensi* mosquitoes and Swiss Webster mice. Sporozoites were collected through infected mosquito's salivary glands dissection.

### Immunization of mice and challenge

Six to eight weeks old female BALB/c mice (Harlan), were used for immunization purposes. Both, PPV-PYCS and recombinant viruses, resuspended in sterile PBS, were injected subcutaneously (s.c). Immunization schedules (dose, route, number of mice) are indicated in Table 1. Non-immunized and immunized mice were challenge 13 days after the booster by inoculation of 5×10^5^ *P. yoelii* sporozoites by the intravenous (i.v) route into the mouse tail vein.

**Table 1:** Dosage of different vaccination regimes: A summary of priming and boosting agents used in the study with the corresponding dosages.

Experiment	Priming		Boosting		Number of mice
	Agent	Dose	Agent	Dose	
**IFN-γ ELISPOT**	VV-PYCS	10^7^ PFU	VV-PYCS	10^7^ PFU	5
	PPV^1^	10 µg	VV-PYCS	10^7^ PFU	5
	PPV	50 µg	VV-PYCS	10^7^ PFU	5
	PPV	10 µg	MVA-PYCS	10^7^ PFU	5
	PPV	50 µg	MVA-PYCS	10^7^ PFU	5
	PPV	100 µg	MVA-PYCS	10^7^ PFU	5
	PPV	10 µg	VV-LUC	10^7^ PFU	5
	–		MVA-PYCS	10^7^ PFU	5
	nrPPV^2^	50 µg	MVA-PYCS	10^7^ PFU	5
	PPV	10 µg	PPV	10 µg	5
	PPV	100 µg	PPV	100 µg	5
**Intra-cellular Cytokine Staining (ICS)**	PPV	50 µg	PPV	50 µg	4
	PPV	50 µg	MVA-PYCS	10^7^ PFU	4
	DNA-PYCS	100 µg	MVA-PYCS	10^7^ PFU	4
	nrPPV	50 µg	nrPPV	50 µg	4
	PBS		PBS		4
**Challenge Studies**	VV-PYCS	10^7^ PFU	VV-PYCS	10^7^ PFU	5
	PPV	10 µg	VV-PYCS	10^7^ PFU	5
	PPV	50 µg	VV-PYCS	10^7^ PFU	5
	PPV	50 µg	MVA-PYCS	10^7^ PFU	5
	FLU-PYCS	10^7^ PFU	MVA-PYCS	10^7^ PFU	5
	PPV	10 µg	PPV	10 µg	5
	–		–		5

All agents were administered s.c except for DNA-PYCS which was injected intradermally.

1 PPV refers to PPV-VLPs containing the CD8 epitope of *P.yoelii* CS protein.

2 nrPPV refers to non-recombinant PPV-VLPs.

### ELISPOT assay

The ELISPOT assay was used to detect epitope-specific IFN-γ-secreting cells [19]. Briefly, nitrocellulose-bottomed 96-well plates were coated with anti-mouse IFN-γ mAb R4-6A2 (8 mg/ml, Pharmingen, San Diego, CA). After overnight incubation at room temperature, wells were washed three times with RPMI 1640, then 100 µl of medium supplemented with 10% FCS were added to each well, and plates incubated at 37°C for 1 h. Triplicate cultures were prepared with serial doubling dilutions of immunized splenocytes, beginning with 10^6^ cells/well. P815 cells (H-2^d^), used as antigen-presenting cells (APC), were pulsed with 10^−6^ M of the synthetic peptide SYVPSAEQI, corresponding to the *P. yoelii* CS protein, and treated with mitomycin C (30 µg/ml, Sigma). After several washes with culture medium, 10^5^ P815 cells were added to each well. Control P815 cells were not pulsed with the peptide. Plates were incubated for 26–28 h at 37°C, washed with PBS containing 0.05% Tween-20 (PBS-T) and incubated overnight at 4°C with biotinylated anti-mouse IFN-γ mAb XMG1.2 (2 µg/ml, Pharmingen) in PBS-T. Plates were washed with PBS-T and peroxidase-labeled avidin (Sigma; 100 µl, 1/800 dilution in PBS-T) was added to each well. One hour later, wells were washed with PBS/T and PBS. Spots were developed by adding 50 mM Tris-HCl, pH 7.5 containing 1 mg/ml of 3,3′-diaminobenzidine tetrahydrochloride (Sigma) and 0.015% H_2_O_2_. When the plates were completely dry, the number of spots was determined with the aid of a stereomicroscope.

### Intra-cellular Cytokine Staining (ICS) Analysis

Multiparameter flow cytometry was performed to study the efficacy of vaccination regimes in altering the magnitude and polyfunctionality of CD8^+^ T-cells, as previously described [32]. Groups of mice (4 per group) were inoculated with different combination of immunogens, VLP-PYCS/VLP-PYCS (50 µg each time), VLP-PYCS (50 µg)/MVA-PYCS (10^7^ pfu), DNA-PYCS (100 µg)/MVA-PYCS (10^7^ pfu), non-recombinant VLP/VLP (50 µg each) and PBS/PBS. Following immunization, the animals were sacrificed after 56 days post-boost to study memory responses. Splenocytes (4×10^6^) from the sacrificed animals were stimulated with 1 µg/ml of CS specific CD8 peptide (SYVPSAEQI) along with Brefeldin (1 µg/ml) (BD Bioscience) for 6 hours in a 96 well plate. After incubation, the cells were washed twice with PBS and stained with LIVE/DEAD fixable dead cell stain kit, following which, the cells were washed and blocked with CD16/CD32 antibody (Fc Block; BD Bioscience). The cells were then stained for surface markers with CD4-Alexa 700, CD8-V500, CD127-V450 and CD62L-FITC (BD Bioscience). This was followed by permeabilisation using BD Cytofix/Cytoperm™ Kit (Becton Dickinson) and staining for intracellular cytokines using IFN-γ PeCy7, TNF-α PE and IL-2 APC. Nearly 50,000 cells were then passed through LSRII flow cytometer (Becton Dickinson). The data generated were analyzed using Flo-Jo (Tree Star. Inc) and SPICE (ver 5.0).

### Quantitation of liver stages

At 42 h following sporozoite challenge, livers were removed and *P. yoelii* liver-stage parasites were measured by quantification of parasite-specific 18S rRNA in total liver RNA as described elsewhere [20]. Livers were homogenized in a Ten Broeck tissue grinder (VWR Scientific) in 4 ml of a denaturing solution (4 M guanidinium thiocyanate, 25 mM sodium citrate [pH 7], 0.5% sarcosyl) made fresh as a working solution (50 ml of denaturing solution, 0.2 M -2-mercaptoethanol). Total liver RNA was then isolated from the liver homogenate by using the TRIzol reagent (Gibco/BRL, Grand Island, N.Y.) as outlined in the product insert. One microgram of RNA was treated with 1.0 U of DNase I (Boehringer Mannheim, Mannheim, Germany) and was then converted to cDNA by the Superscript preamplification system for first-strand cDNA synthesis (Gibco/BRL), using random hexamers in a 21-µl total volume as outlined in the product insert.

For quantification of parasite-specific rRNA by quantitative-competitive reverse transcription-PCR (RT-PCR)[21], parasite-specific rRNA was amplified from 5 µl of the cDNA mixture in a PCR master mix containing 46 µl of PCR Supermix (Gibco/BRL), 1 µl each of parasite-specific primers PB1 and PB2 (1) (12 µM, final concentration), 0.2 µl (1 U) of Taq DNA polymerase (Sigma, St. Louis, Mo.), and 1 µl of a known concentration of competitor plasmid. Then 35 cycles of amplification in a PCR Express (Hybaid, Middlesex, United Kingdom) thermocycler were performed under the following conditions: 94°C for 1 min, 60°C for 2 min, and 72°C for 1 min. An initial denaturation step at 94°C for 2 min and a terminal elongation step at 72°C for 10 min were also included. Target and competitor amplicons were resolved on ethidium bromide-stained 2% agarose gels and photographed by the Eagle Eye II still video system (Stratagene, La Jolla, California), and the image was stored electronically. Target-to-competitor ratios were then determined using the NIH Image software program. We performed a series of amplifications with different competitor concentrations and used target-to-competitor ratios from each competitor concentration in linear regression analysis to determine the competitor concentration where target and competitor amplicon ratios were equivalent. This concentration was used as a relative measure of liver parasite burden. Equal cDNA synthesis between samples was ensured by amplification of the housekeeping ß-actin gene at PCR conditions below saturation.

### Statistical analysis

Statistical analysis of ELISPOT and protection assays was performed by a one-way ANOVA with Tukey post test and Bonferroni correction to all groups. For the statistical analysis of ICS data we used a novel approach that corrects measurements for the medium response (RPMI) and at the same time allows the calculation of confidence intervals and p-values of hypothesis tests [33]. The background for the different cytokines in the unstimulated controls never exceeded 0.05%. The data analysis program, Simplified Presentation of Incredibly Complex Evaluations (SPICE, version 4.1.5, Mario Roederer, Vaccine Research Center, NIAID, NIH), was used to analyze and generate graphical representations of T cell responses detected by polychromatic flow cytometry. All values used for analyzing proportionate representation of responses are background-subtracted. In all cases, P values of less than 0.05 were considered statistically significant. All statistic tests were performed using GraphPad Prism version 4.03 for Windows (GraphPad Software, San Diego, C).
